# Size-controllable Ni_5_TiO_7_ nanowires as promising catalysts for CO oxidation

**DOI:** 10.1038/srep14330

**Published:** 2015-09-23

**Authors:** Yanan Jiang, Baodan Liu, Lini Yang, Bing Yang, Xiaoyuan Liu, Lusheng Liu, Christian Weimer, Xin jiang

**Affiliations:** 1 Shenyang National Laboratory for Materials Science, Institute of Metal Research (IMR), Chinese Academy of Sciences (CAS), No. 72 Wenhua Road, Shenyang 110016 China; 2 College of Chemistry, Liaoning University, Shenyang, Liaoning, 110036, China; 3 Institute of Materials Engineering, University of Siegen, Paul-Bonatz-Straße 9-11, Siegen, 57076 Germany

## Abstract

Ni_5_TiO_7_ nanowires with controllable sizes are synthesized using PEO method combined with impregnation and annealing at 1050^o^C in air, with adjustment of different concentrations of impregnating solution to control the dimension of nanowires. The resulting nanowires are characterized in details using X-ray diffraction, scanning electron microscopy, transmission electron microscopy and energy dispersive X-ray analysis. In addition, the CO catalytic oxidation performance of the Ni_5_TiO_7_ nanowires is investigated using a fixed-bed quartz tubular reactor and an on-line gas chromatography system, indicating that the activity of this catalytic system for CO oxidation is a strong dependency upon the nanocrystal size.When the size of the Ni_5_TiO_7_ nanowires is induced from 4 μm to 50 nm, the corresponding maximum conversion temperature is lowered by ~100 ^o^C.

The climate debate in the context of exhaust pollution has been paid extensive attention worldwide for many years because of jeopardizing human health, especially the CO emission from automobile exhaust and incomplete combustion of hydrocarbons has been regarded as a major problem^1^,^2^. As a result, the CO conversion and removal using efficient catalysts has been a hot topic either in fundamental research or industrial applications. Over the past decades, metal oxide compounds decorated with noble metal particles such as Pt/SnO_2_, Pd/SnO_2_, Au/TiO_2_ etc have been demonstrated to be efficient catalysts for CO oxidation^3^,^4^. However, the limited resource and high cost of noble metals promote us to search for alternative peers with comparable catalytic capability and commercially available low-cost. With this demand, extensive efforts are being focused on the developing of highly efficient and stable, but cost down metal oxide catalysts free of noble metal decorations for CO oxidation.

So far, research on transition metal oxides as catalysts for CO oxidation has made significant progress based on the combination of different metal compound oxides. For example, some composite metal oxides such as CuO_x_/CeO_2_, CoO_x_/Al_2_O_3_, MnO_x_/Al_2_O_3_, Ce_1-x_Ti_x_O_2_, Ce_0.8_Zr_0.2_O_2_ have been identified as good catalysts for CO oxidation^5^,^6^,^7^,^8^,^9^. It’s well known that the catalytic activity of transition metal oxides is strongly dependent on some key factors such as well-defined facets, size, morphology, composition and crystallinity. Especially, the smaller size directly results in the huge increase of specific surface area and active sites on the surface and thus leads to amazing performance enhancement^10^,^11^. Therefore, it is quite essential to develop a facile approach for producing transitional metal oxide catalysts with large yield, controllable size and dimension, tunable catalysis performance and low cost considering the future industrial application. In contrast to conventional popular methods for synthesizing transition metal oxides, for example, chemical vapour deposition (CVD)^12^, solvothermal synthesis^13^, and hydrothermal synthesis^14^, the innovation approach of Plasma Electrolytic Oxidation (PEO) has been demonstrated extremely successful and efficient in producing porous MO_x_/TiO_2_/Ti oxide nanostructures (where M = Ni, Cu, Mn, W, Co) and (CeO_x_, ZrO_2_)/TiO_2_/Ti coatings^15^, with its obvious advantages of an easy operation, low-cost and versatility in dimension controlling and composition tailoring^16^.

Ni_5_TiO_7_, a promising catalyst for tar conversion, was first synthesized with flux method by Fumio Shimura and Tsutomu Kawamura in 1976^17^,^18^. It possesses an orthorhombic symmetry with a unit cell lattice constants a = 9.20 ± 0.02 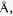 b = 2.99 ± 0.01 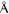 and c = 12.17 ± 0.04 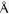
17. The structural formula of Ni_5_TiO_7_ can be simply regarded as the combination of NiO and TiO_2_ with a stoichiometric ratio of NiO:TiO_2_ = 5:1 (or Ni:Ti:O = 5:1:7). However, the formation of Ni_5_TiO_7_ crystal cannot be achieved by simply heating the NiO and TiO_2_ powders at high temperature. Until very recently, Jiang and his co-workers in Siegen University (Germany) reported the fabrication of nanostructured Ni_5_TiO_7_ catalyst using PEO method and demonstrated its outstanding performance in biomass gasification process^18^. However, no further work regarding to the size controlling of Ni_5_TiO_7_ nanocatalyst, which is crucial for understanding its nucleation, crystallization and further catalytic performance improvement, was preceded. In addition, exploring its multi-functions in environment-related issues and long-term stability is still essential for considering its future practical applications in environment processing.

In this context, the size controlling of Ni_5_TiO_7_ catalysts fabricated by PEO method is systemically investigated through tailoring the concentration of Ni ion in impregnation solutions. The morphology and size evolution of Ni_5_TiO_7_ nanostructures as a dependence of Ni ions are also discussed. As a key part, the CO catalytic oxidation behaviour of the as-prepared Ni_5_TiO_7_ nanowires used as catalysts is also evaluated for exploring their promising applications in CO conversion. In addition, the size influence of Ni_5_TiO_7_ catalysts on their CO catalytic efficiency and the stability at high temperature are also studied. It is demonstrated that the nanoscaled Ni_5_TiO_7_ catalysts with huge surface area and excellent crystallinity exhibit broad application potential and incomparable advantage in CO oxidation and biomass gasification, especially in car exhaust processing or the degradation of waste gas pollutions, for the sake of their high catalytic performance, low cost and easy preparation process.

## Results and Discussion

**Figure 1:**
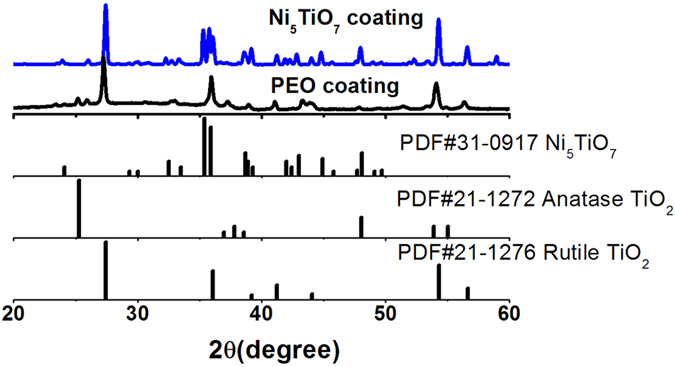
XRD patterns of PEO coating and Ni_5_TiO_7_ layer after annealing at 1050    °C for 1 hour; Standard XRD data of Ni_5_TiO_7_, anatase TiO_2_ and rutile TiO_2_ are also shown as references.

X-ray diffraction (XRD) is first used to examine the crystalline structures and phase purities of as-synthesized samples obtained at PEO stage and subsequent annealing. Figure 1 shows the XRD patterns of PEO coating and its subsequent dipping into 0.5 M Ni(NO_3_)_2_ solution and annealing at 1050 ^o^C . It is found that mixed anatase and rutile TiO_2_ phases have been directly formed in PEO coating after the arc-discharging process in electrolyte. The peak from Ti substrate is not observed because of the excessive thickness of PEO coating (~10 μm). In addition, a dome peak in the range of 20–40^o^ is also observed, which indicates the formation of some amorphous phase. After dipping the PEO coating in Ni(NO_3_)_2_ solution for 1 hour and heated at 1050 ^o^C, Ni_5_TiO_7_ phase with good crystallinity appears in the surface of the PEO coating and shows predominant diffraction intensity (Fig. 1), in good agreement with the standard structure data of Ni_5_TiO_7_ crystal (No. JCPDS: 31-0927). Meanwhile, the preformed anatase TiO_2_ phase has completely disappeared from the PEO coating and only rutile TiO_2_ phase is maintained due to the metastable feature of anatase TiO_2_ at a critical phase transition temperature in the range of 400–1050 ^o^C.^23^ The formation of Ni_5_TiO_7_ phase implies that Ni ions incorporated into porous PEO surface/matrix have reacted with Ti/TiO_2_ at high temperature.

**Figure 2:**
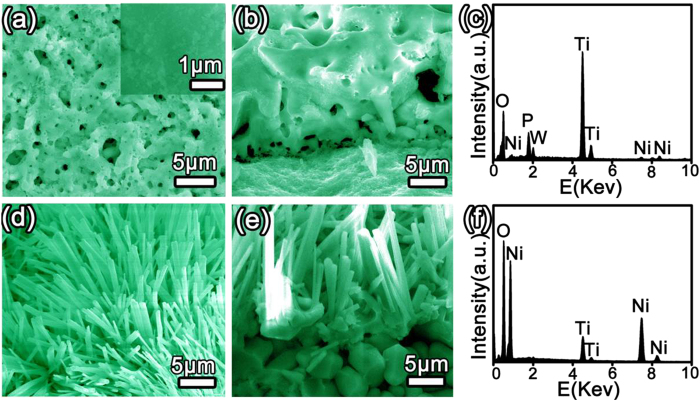
(**a**,**b**) SEM surface and cross-sectional morphologies of PEO coating; (**d**,**e**) SEM surface and cross-section morphologies of Ni_5_TiO_7_ grown on PEO coating at 1050 ^o^C; (**c**,**f**) EDS results of PEO coating and Ni_5_TiO_7_, respectively.

Figure 2 shows the morphologies and compositions of the PEO coating and the as-synthesized Ni_5_TiO_7_ nanostructures annealed at 1050 ^o^C. It’s found that the PEO coating with a thickness of 10 μm has a rough morphology with concaved pores uniformly distributing on the surface (Fig. 2a,b). The pores are formed during micro-arc discharging under high voltage and high surface temperature, which produces reduplicate melt and solidification reactions on Ti surface. Composition analysis performed on the PEO coating shows that the layer is mainly made of Ti and O elements (TiO_2_) with Ni, P and W etc as minorities (Fig. 2c), indicating the formation of porous TiO_2_ film. These Ni, P and W elements are directly from the electrolytes we used (see experimental section). The detection of Ni etc elements in the PEO coating suggests that the amorphous phase corresponding to the broad dome in XRD pattern (Fig. 1) may be assigned to some Ni-containing compound. To obtain Ni_5_TiO_7_ nanocrystals, the porous PEO coating dipped into Ni(NO_3_)_2_ solution is annealed at 1050 ^o^C for 1 hour, and it is found that needle-like nanowires with diameters ranging from 100 nm to 1 μm are densely covered on the PEO coating surface (Fig. 2d). Cross-section SEM observation presented in Fig. 2e clearly shows that these nanowires are directly grown from the top layer of the porous coating and are tightly combined with the coating. In contrast to the predominant Ti and O constituents in initial PEO coating, the chemical compositions detected from the local nanowires are identified as Ni, Ti and O with a stoichiometric ratio close to Ni_5_TiO_7_ (Fig. 2f), further demonstrating the formation of Ni_5_TiO_7_ phase after high-temperature annealing.

**Figure 3:**
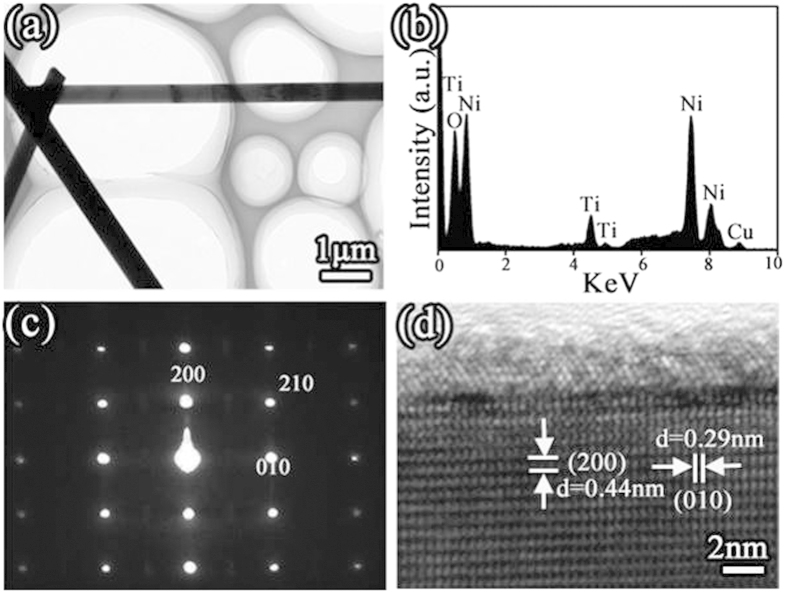
(**a**) representative TEM image of Ni_5_TiO_7_ nanowires by annealing PEO coating at 1050 ^o^C; (**b**) EDS spectrum recorded from the nanowire; (**c**) SAED pattern taken from Ni_5_TiO_7_ nanowire and (**d**) its corresponding HRTEM image.

Figure 3a shows a representative TEM image of Ni_5_TiO_7_ nanowires transferred from the porous TiO_2_ coating. The smoothness of nanowire surface and the uniformity of nanowire diameter in the range of 200–500 nm can be clearly confirmed. No particles in nano-scaled size can be found at the nanowire surface and tip-end. Careful EDS analyses performed on different positions of the nanowire conclude that the nanowires are indeed made of Ni, Ti and O with an atomic ratio of Ni:Ti:O = 5:1:7 (Fig. 3b and Fig. S1), matching well with the composition value of standard Ni_5_TiO_7_ crystal. The Cu peak with negligible signal is directly from the Cu TEM grid for supporting the samples. Absence of any other impurity peak related to P and W elements that are introduced during PEO process demonstrates again the high phase and chemical purity of Ni_5_TiO_7_ nanowires. The high-temperature annealing of PEO coating at 1050 °C in ambient environment only leads to the nucleation and crystallization of Ni_5_TiO_7_ phase and does not introduce any possible contamination. The superior crystallinity of Ni_5_TiO_7_ nanowires, proved by the succinct selective area electron diffraction (SAED) pattern (Fig. 3c) and HRTEM data (Fig. 3d), verify the single crystalline nature of Ni_5_TiO_7_ nanowires. The separated and periodic diffraction spots and well-aligned atom ordering confirm that Ni_5_TiO_7_ nanowires prepared by PEO method are free of structural defects like microtwins and stacking faults, indicating the obvious advance of this technique in the crystallinity controlling in comparison with other methods for metal oxide nanostructure preparation^19^. The lattice distance between two adjacent lattice planes parallel and perpendicular to the growth direction are measured to be 0.44 nm and 0.29 nm, respectively, matching well with the d-spacing value of (200) and (010) planes of orthorhombic Ni_5_TiO_7_ crystal. The growth orientation of Ni_5_TiO_7_ nanowires, along [010] direction, can also be confirmed as a result of a strong growth competition of the (010) surface against other low-indexed planes of the side facets. In addition, an outer layer with a thickness of few nanometers can be found on the surface of the Ni_5_TiO_7_ nanowire.

**Figure 4:**
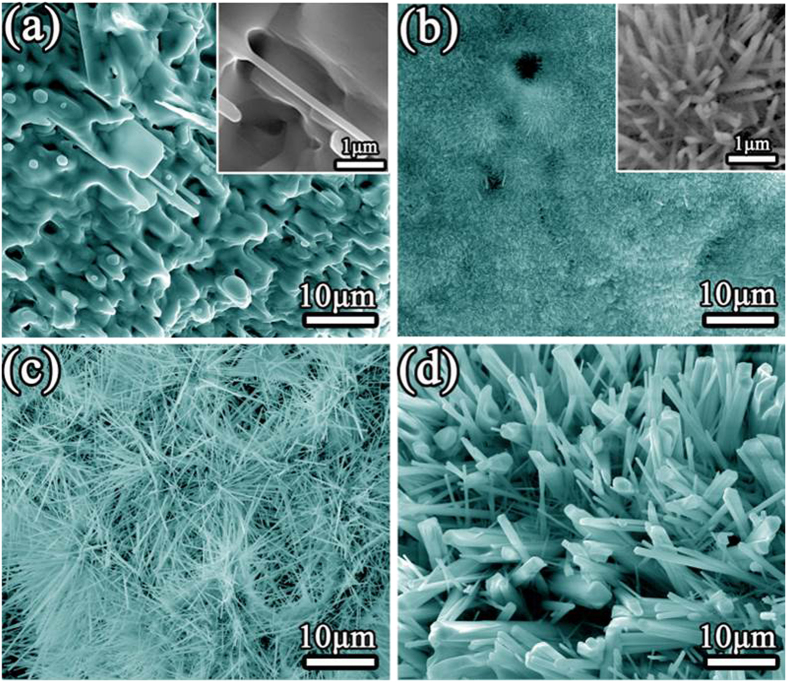
SEM morphologies of Ni_5_TiO_7_ nanowires grown on PEO coating in different Ni(NO_3_)_2_ concentrations of impregnation solutions at 1050 ^o^C: (a) without dipping; (b) 0.1 M; (c) 0.5M and (d) 2 M.

In the synthesis of Ni_5_TiO_7_ nanowires, it is found that Ni ions in the impregnation solution play a key role in organizing the initial nucleation locations and are essential for the nucleation (or growth) density tailoring of Ni_5_TiO_7_ nanowires. In order to investigate the influence of Ni ion concentrations on the size and morphology evolution of Ni_5_TiO_7_ nanowires, the PEO coatings are dipped into Ni(NO_3_)_2_ solutions with a concentration of 0.1M, 0.5 M and 2M for 1 hour, respectively. Figure 4 shows the typical morphology of Ni_5_TiO_7_ nanowires evolved from as-prepared PEO coating and pre-treated samples annealed at 1050 ^o^C for 1h. Notably, a significant difference in the morphology and size of Ni_5_TiO_7_ nanowires can be distinguished from these four samples. When the PEO coating is annealed without dipping into Ni(NO_3_)_2_ solution, only a small fraction of nanowires and nanoparticles with strip shapes and huge size can be found on its surface, as shown in Fig. 4a. XRD and EDS results (Supporting Information Figure S2) clearly demonstrate that the nanowires are Ni_5_TiO_7_, suggesting that Ni ions in the electrolyte has been absorbed/stored in the PEO coating during arc-discharging process and further reacted with Ti/TiO_2_ to lead to the formation of Ni_5_TiO_7_ phase. Figure 4b–d show the SEM images of Ni_5_TiO_7_ nanowires corresponding to Ni(NO_3_)_2_ solution concentrations of 0.1M, 0.5 M and 2M, respectively and annealed at 1050 ^o^C for 1 hour. It can be found that the dipping process has a significant effect on the morphology and size evolutions of the Ni_5_TiO_7_ nanowires. Apparently, impregnating the PEO coating with 0.1M Ni(NO_3_)_2_ solution results in the drastic size reduction of Ni_5_TiO_7_ nanowires from 2–5 μm for as-prepared one to an average diameter of 50 nm (Fig. 4b). Inset of Fig. 4b gives a clear morphology and dimension size of Ni_5_TiO_7_ nanowires protruding from the PEO coating surface. When the Ni concentration is increased to 0.5 M, both the diameter and length of Ni_5_TiO_7_ nanowires have evolved to ~300 nm and ~10 μm correspondingly, as shown in Fig. 4c. Further increase of the Ni concentration to 2M directly induces the fast growth of Ni_5_TiO_7_ nanowires to an average diameter of 4 μm (Fig. 4d). All these SEM observations clearly demonstrate that the dipping process is of great significance in tailoring the morphology and general size of Ni_5_TiO_7_ nanowires. Consequently, the morphology and size of Ni_5_TiO_7_ nanowires can be correspondingly modified by controlling the concentrations of Ni(NO_3_)_2_ solution. It is reasonable to understand the morphology and size evolution of Ni_5_TiO_7_ nanowires as a dependence of Ni(NO_3_)_2_ solution concentrations. In the case of lower content of Ni(NO_3_)_2_ solution, a thin layer of Ni(NO_3_)_2_ or Ni will cover on the PEO coating surface and aggregate into small islands under high-temperature annealing. These tiny Ni-containing islands with higher surface energy are preferential nucleation sites for the initial nucleation of Ni_5_TiO_7_ phase and serve as the seeds for controlling their subsequent crystallization. It should be strengthened that the initial size of these Ni-containing nanoparticles is of crucial importance in dominating the size of Ni_5_TiO_7_ nanowires. With the concentration increase of the Ni(NO_3_)_2_ solution, more Ni(NO_3_)_2_ or Ni will be deposited on the PEO coating surface and will give rise to the corresponding thickness enhancement of Ni-containing film. As a result, the nanoislands evolved from the thin film will correspondingly possess a large size for guiding the nucleation and the growth of Ni_5_TiO_7_ nanowires. The same phenomenon and process can also be expected in the case of 2M Ni(NO_3_)_2_ solution for producing the largest size of Ni_5_TiO_7_ nanowires. The feasible size-controlling of Ni_5_TiO_7_ nanowires will undoubtedly provide more space for their catalytic performance tailoring and open up more opportunities for their applications.

Based on detailed SEM observations and TEM characterizations, a tentative reaction mechanism describing the formations of the PEO oxide coating and the Ni_5_TiO_7_ nanowires is proposed^20^,^21^. At the first stage of PEO process, the titanium will be charged to Ti ions and its surrounding aqueous solution (mainly H_2_O) will be decomposed into H^+^ and OH^–^ under high voltage and temperature, as described below^22^:


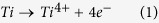



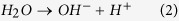


Following this step, the Ti^4+^ will react with OH^-^ to lead to the formation of anatase and rutile TiO_2_ phases under a huge temperature gradient generated by micro-arc discharge and aqueous cooling. The formation reaction of TiO_2_ can be described as follows^23^:


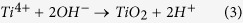


At the third stage of PEO process, Ni(CH_3_COO)_2_ in the electrolyte can be ionized to Ni^2+^ and (CH_3_COO)^–^ by the energetic arc discharge. The Ni^2+^ ions will react with other ions such as Ti^4+^ and OH^-^ under high temperature and high voltage, which leads to the formation of Ni-containing phase (like amorphous Ni_5_TiO_7_) in the PEO coating, similar to the formation of CaTiO_3_ produced by the PEO method^24^,^25^. It should be noted that the assertion of the formation of amorphous Ni-containing phase can be deduced from the dome peak in the XRD pattern (Fig. 1), which results from the repeatedly heating and cooling processes under high surface temperature produced by the micro-arc discharge in the electrolytic solution^26^. It has been reported that the momentary temperature in the local discharge zones can be up to 4000~8000 K in a short time^26^. When the PEO coating is subject to a high-temperature annealing at 1050 ^o^C, the Ni-contained amorphous phase will crystallize and the other excessive Ni ions involved in the porous coating layer will diffuse into the top layer to provide Ni precursor and participate in the reaction with Ti/TiO_2_ and oxygen. Finally, Ni_5_TiO_7_ nanostructures with high density can be achieved.

**Figure 5:**
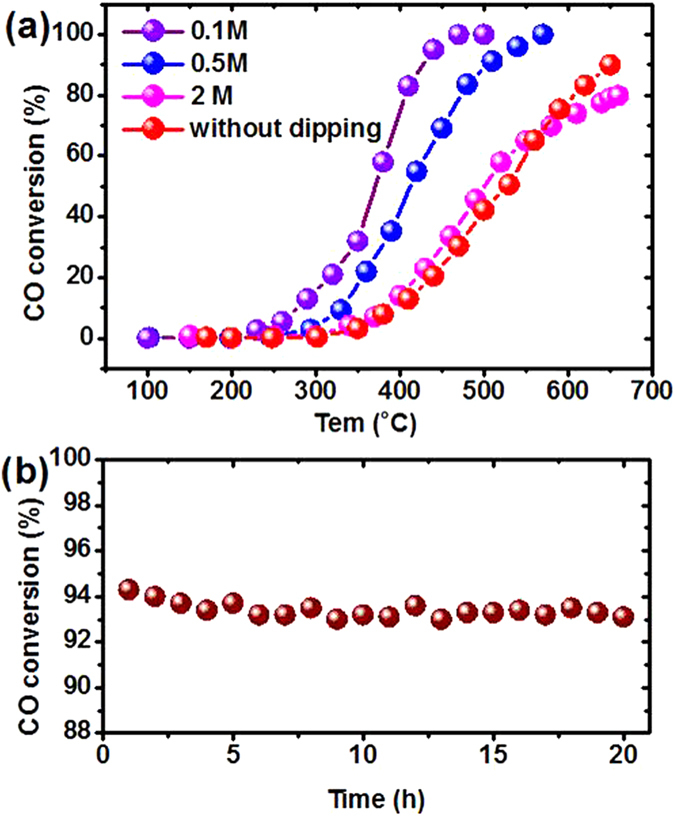
(a) Temperature-dependent CO catalytic oxidation performance over Ni_5_TiO_7_ nanowires catalysts prepared in different concentrations of impregnating solution; (b) the long-term stability test of Ni_5_TiO_7_ nanowires at 440 ^o^C .

Previous study has demonstrated the outstanding performance of Ni_5_TiO_7_ catalysts in tar conversion with naphthalene as the target sample^18^. For considering its potential application in car exhaust processing, the CO oxidation over the as-prepared Ni_5_TiO_7_ nanowires is also evaluated. In order to characterize the “real” catalytic performance of the catalysts, Ni_5_TiO_7_ nanowires attached on TiO_2_ coatings are peeled off from the Ti substrate. On the other hand, the Ni_5_TiO_7_ nanowires in the areas with uniform morphology and high growth density are used for the CO evaluation test in order to depress the influence of porous TiO_2_ coating to the greatest extent. Figure 5a summarizes the CO conversion rate as a dependence of reaction temperature for all Ni_5_TiO_7_/TiO_2_ composite catalysts prepared under different concentrations of Ni(NO_3_)_2_ solutions. It is noticed that the Ni_5_TiO_7_ nanowires corresponding to a 0.1M Ni(NO_3_)_2_ solution exhibit the best CO conversion capability in comparison with the other samples. Considering the morphology or growth density dependence on the concentration of Ni(NO_3_)_2_ solution, it is found that the Ni_5_TiO_7_ nanowires corresponding to 0.1M Ni(NO_3_)_2_ solution have the smallest nanowire size and largest growth density than the other samples, indicating that the CO conversion efficiency of Ni_5_TiO_7_ nanowires depends strongly on their size and growth density (Fig. 4). From Fig. 5a, it can be seen that the start temperature for CO conversion for Ni_5_TiO_7_ nanowires in the case of 0.1M Ni(NO_3_)_2_ solution is around 230 ^o^C. This critical conversion temperature is still higher than that of metal or metal oxide like NiO/Au^27^, Pt/CeO_2_^2^, but it should be kept in mind that the catalytic reaction of Ni_5_TiO_7_ nanowires is performed without any assistance of highly efficient noble metal catalysts. When the reaction temperature is increased to 440 ^o^C, the CO oxidation capability is enhanced sharply and the catalytic activity approaches to the maximum. Almost all the CO has been completely converted into CO_2_. For Ni_5_TiO_7_ nanowires corresponding to a Ni(NO_3_)_2_ concentration of 0.5 M, they exhibit the second best catalytic performance in all the four samples, which shows a starting reaction temperature at 300 ^o^C and an end conversion temperature at 550 ^o^C. However, the Ni_5_TiO_7_ nanowires corresponding to 2M Ni(NO_3_)_2_ solution show quite similar/comparable catalytic performance to the sample without any dipping. The significant difference in catalytic performance undoubtedly comes from the discrepancy of Ni_5_TiO_7_ nanowires in size and growth density, which provides different active sites for CO catalytic reaction. From above results, it can be concluded that the catalytic properties of Ni_5_TiO_7_ nanowires have close relation to their morphology and surface. SEM observation shown in Fig. 4 has demonstrated that increasing the concentrations of Ni(NO_3_)_2_ solutions can lead to their size increase correspondingly, directly resulting in the weakening of the CO conversion rate and performance. Like most metal oxide catalysts, the CO catalytic oxidation of Ni_5_TiO_7_ nanowires is closely related to nanowire size, the exposed surface area, mean diameter and the contact structure between the catalyst and the support metal oxides^28^. It is believed that the desorption of CO and O_2_ on the surface of Ni_5_TiO_7_ nanowires will react each other on the effect of Ni-O bonds and results in the catalytic oxidation^29^. In addition, the evaluation of the long-term stability of Ni_5_TiO_7_ nanowires is also carried out. Figure 5b shows the continuous CO conversion test curve of Ni_5_TiO_7_ nanowires, which is annealed at 1050 ^o^C and corresponding to a 0.1M Ni(NO_3_)_2_ solution. The long-term stability test is carried out at 440 ^o^C since it shows the best activity in CO oxidation (Fig. 5a). Notably, the Ni_5_TiO_7_ nanowires exhibit extremely stable catalytic performance and no degradation of CO conversion efficiency is observed in the 20-hour test, further demonstrating the excellent high-temperature adaptation ability and thus it can be compared with some metal oxide catalysts like Co_3_O_4_ and NiCo_2_O_4_^30^,^31^.

**Figure 6:**
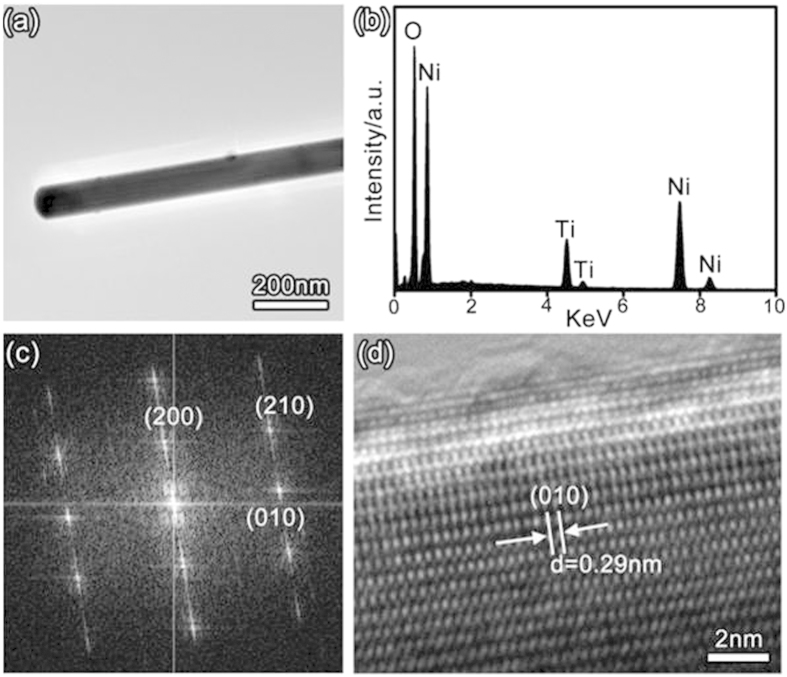
(a) Representative TEM image of Ni_5_TiO_7_ nanowires after CO catalytic oxidation; (b) EDS spectrum recorded from the nanowire; (c) FFT pattern taken from Ni_5_TiO_7_ nanowire and (d) its corresponding HRTEM image.

To comprehensively evaluate the application potential of Ni_5_TiO_7_ nanowire catalysts, the structural stability of Ni_5_TiO_7_ nanowires after several cycle high-temperature catalysis tests is investigated. The Ni_5_TiO_7_ nanowires continuously working at 440 ^o^C for 20 hours are used as the samples for structure and crystallinity evaluations using TEM, as shown in Fig. 6. It is found that the morphology and appearance of the nanowires are not destroyed after the catalytic reaction (Fig. 6a). The nanowires still maintain their initial surface smoothness as the one shown in Fig. 3a. Further TEM observation on high-magnifications reveals that the nanowires still show superior crystallinity and the ordering of atoms is not deteriorated (Fig. 6d). Especially, the outer surface of the Ni_5_TiO_7_ nanowires still keeps the same feature as the one before catalytic reaction. Absence of any nanoparticles on the nanowire surface implies that the nanowires are not decomposed or involved into any phase transition reactions. Additionally, we do not observe any clues that verify the formation of polycrystalline or amorphous phases in the Fast Fourier Transition (FFT) (Fig. 6c). Composition analysis using high-resolution EDS confirms the same constitutions as the as-synthesized nanowires before catalytic reaction and impurity peaks from any possible phases are not found (Fig. 6b), further demonstrating the chemical stability of our nanowires involved in the CO oxidation. Apart from these merits, it should be strengthened that the Ni_5_TiO_7_ nanowires are *in-situ* fabricated on porous TiO_2_ surface with improved substrate adherence. This peculiar advantage will in turn promote their direct fabrications on honeycomb-like metal substrate using PEO method and accelerate their practical utilization. With its predominant advantages in low-cost and easy synthesis process, as well as the superior performance in CO conversion and biomass gasification, the Ni_5_TiO_7_ nanowires will certainly open up more space as an efficient and chemically/structurally stable catalyst for environment-friendly applications ranging from car exhaust processing to chemical pollutant cleaning.

## Conclusion

In summary, Ni_5_TiO_7_ nanowires with single crystal feature have been synthesized using conventional PEO method with subsequent impregnation and annealing processes. The size of Ni_5_TiO_7_ nanowires can be selectively tailored through simply controlling the Ni(NO_3_)_2_ concentration in dipping solution and an average minimized diameter as small as 50 nm can be obtained. CO oxidation tests demonstrates that the catalytic performance of Ni_5_TiO_7_ nanowires is strongly dependent on their size and growth density and the Ni_5_TiO_7_ nanowires with an average diameter of 50 nm show the best CO conversion efficiency. Long-term catalytic evaluation with a total reaction time of 20 hours and the precise HRTEM analysis verify the superior stability in catalytic performance and microstructure, which allows the current Ni_5_TiO_7_ nanowires as promising catalysts for diverse applications ranging from biomass gasification to car exhaust process.

## Experimental Section

### Samples preparation

Commercially available pure grade I titanium sheets (10mm × 10mm × 1mm, Shanxi Baotai group) and graphite were connected to the positive and negative poles of power supply as anode and cathode, respectively. Titanium sheets are also used as the substrates to form porous TiO_2_ coatings and the Ti precursor. Before the PEO process, the Ti substrates are ground using #1000 sand paper to remove the contaminated surface (TiO_2_ or other impurity) and are cleaned with acetone, ethyl alcohol and distilled water. To promote the PEO process, mixed solutions comprising of sodium phosphate (Na_3_PO_4_·12H_2_O, merck), sodium borate (Na_2_B_4_O_7_·10H_2_O, Merck), sodium tungstate (Na_2_WO_4_·2H_2_O, Merck) and nickel acetate (Ni(CH_3_COO)_2_·4H_2_O, Merck) are stored in a plastic box and are utilized as electrolytes and Ni precursors. It has been reported that PO_4_^3-^, WO_4_^2-^ and B_4_O_7_^2-^ ions can promote a uniform micro-arc generation, which is essential for producing homogeneous PEO coating^32^,^33^. A detailed experimental setup for the PEO reaction can be found in Figure S3. The PEO process was carried out in a fixed applied current density mode of 0.1 A cm^−2^. Other parameters including PEO time and pulse frequency were set at 10 min and 1000 Hz, respectively, for all samples after several rounds optimization. The electrolyte temperature was controlled under 50 ^o^C for a full PEO reaction through circulating water cooling system during the PEO process. After the completion of the porous PEO coating, it was dipped into Ni(NO_3_)_2_ solutions with different concentrations for 1 hour for improving the Ni ion concentrations in the PEO coating. Following this step, the PEO coating were transferred to a resistance furnace for thermal annealing to promote the nucleation and crystallization of Ni_5_TiO_7_ catalysts. After maintaining the reaction at 1050 ^o^C for 1 hour, high density of Ni_5_TiO_7_ nanostructures featured with a wire-like morphology on the surface of PEO coating were obtained.

### Structure and composition characterization

Phase identification of the PEO coating and Ni_5_TiO_7_ nanostructures was examined by an X-ray diffractions (XRD, Rigaku D/max 2400) using Cuk_α_ (λ_kα_ = 0.154056 nm) as the x-ray source over a 2θ angel of 20–60^o^. The morphology of the coatings and Ni_5_TiO_7_ nanostructures are characterized by a field-emission scanning electron microscopy (FE-SEM, FEI Inspect F50) equipped with a Quanta 600 Energy Dispersed X-ray spectrometer (EDS) system. The microstructure, crystallinity and elemental compositions of Ni_5_TiO_7_ nanowires scratched from the coating surfaces were characterized by a transmission electron microscopy (TEM, Tecnai, F20) under an accelerated voltage of 200 kV.

### Catalytic tests

All the catalytic activity evaluations of Ni_5_TiO_7_/TiO_2_ composite catalysts for CO oxidation were carried out by using a fixed-bed quartz tubular reactor. The catalysts peeled off from the Ti substrate were placed in the active zone of the quartz tubular reactor. Mixed gases (1.0% CO, 10% O_2_, balanced with helium) are transported into the reactor at a flowing rate of 10 ml/min. The reactants and products were analyzed by using an on-line gas chromatography system (Agilent 7890A) with a molecular sieve column. CO and CO_2_ gases were detected with a thermal conductivity detector. All the catalytic tests were carried out at the temperatures ranging from 25 ^o^C to 650 ^o^C.

## Additional Information

**How to cite this article**: Jiang, Y. *et al.* Size-controllable Ni_5_TiO_7_ nanowires as promising catalysts for CO oxidation. *Sci. Rep.* **5**, 14330; doi: 10.1038/srep14330 (2015).

## Supplementary Material

Supplementary Information

## References

[b1] Shelef M. & McCabe R. W. Twenty-five years after introduction of automotive catalysts: what next? Catal. Today 62, 35–50 (2000).

[b2] Bera P., Patil K. C., Jayaram V., Subbanna G. N. & Hegde M. S. Ionic Dispersion of Pt and Pd on CeO_2_ by Combustion Method: Effect of Metal–Ceria Interaction on Catalytic Activities for NO Reduction and CO and Hydrocarbon Oxidation. J Catal 196, 293–301 (2000).

[b3] Saberi M. H., Mortazavi Y. & Khodadadi A. A. Dual selective Pt/SnO_2_ sensor to CO and propane in exhaust gases of gasoline engines using Pt/LaFeO_3_ filter. Sens. Actuators B: Chem. 206, 617–623 (2015).

[b4] Remediakis I. N., Lopez N. & NФrskov J. K. CO Oxidation on Rutile-Supported Au Nanoparticles. Angew. Chem. 117, 1858–1826 (2005). 10.1002/anie.200461699 15712248

[b5] Laguna O. H., Hernandez W. Y., Arzamendi G., Gandia L. M., Centeno M. A. & Odriozola J. A. Gold supported on CuOx/CeO2 catalyst for the purification of hydrogen by the CO preferential oxidation reaction (PROX). Fuel. 118, 176–185 (2014).

[b6] Cao J. L., Wang Y., Zhang T. Y., Wu S. H. & Yuan Z. Y. Preparation, characterization and catalytic behavior of nanostructured mesoporous CuO/Ce_0.8_Zr_0.2_O_2_ catalysts for low-temperature CO oxidation. Appl Catal B-environ 78, 120–128 (2008).

[b7] Wu Z. W. *et al.* Preferential oxidation of CO in H_2_-rich stream over CuO/Ce_1−x_Ti_x_O_2_ catalysts. Appl Catal B: Environl 98, 204–212 (2010).

[b8] Zou Z. Q., Meng M., Guo L. H. & Zha Y. Q. Synthesis and characterization of CuO/Ce_1−x_Ti_x_O_2_ catalysts used for low-temperature CO oxidation. J Hazard Mater 163, 835–842 (2009). 18718718 10.1016/j.jhazmat.2008.07.035

[b9] Konova P., Stoyanova M., Naydenov A., Christoskova S. & Mehandjiev D. Catalytic oxidation of VOCs and CO by ozone over alumina supported cobalt oxide. Appl Catal A: Gen 298, 109–114 (2006).

[b10] Liu S. Q., Tang Z.-R., Sun Y. G., Colmenare Juan Carlos & Xu Y.–J. One-dimension-based spatially ordered architectures for solar energy conversion. Chem Soc Rev 44, 5053–5075 (2015). 25856797 10.1039/c4cs00408f

[b11] Weng B. S., Liu S. Q., Tang Z.-R. & Xu Y.-J. One-dimensional nanostructure based materials for versatile photocatalytic applications. RSC Adv. 4, 12685–12700 (2014).

[b12] Yoshida S. *et al.* Analysis of xanes for indentication of highly dispersed transition mtal oxides on supports. Catal Lett. 12, 277–286 (1992).

[b13] Chang X. T., Sun S. B., Xu X. & Li S. J. Synthesis of transition metal-doped tungsten oxide nanostructures and their optical properties. Mater Lett 65, 1710–1712 (2011).

[b14] Siriwong P., Thongtem T., Phuruangrat A. & Thongtem S. Hydrothermal synthesis, characterization, and optical properties of wolframite ZnWO_4_ nanorods. CrystEngComm 13, 1564–1569 (2011).

[b15] Patcas F. & Krysmann W. Efficient catalysts with controlled porous structure obtained by anodic oxidation under spark-discharge. Appl Catal A: Gen 316, 240–249 (2007).

[b16] Bayati M. R., Molaei R., Moshfegh A. Z., Golestani-Fard F. A strategy for single-step elaboration of V_2_O_5_-grafted TiO_2_ nanostructured photocatalysts with evenly distributed pores. J Alloys Compd. 509, 6236–6241 (2011).

[b17] Shimura F. & Kawamura T. Crystal-Growth of a New Phase Ni_5_TiO_7_ and Its Crystallographic Properties. Jpn J Appl Phys 15, 1403–1404 (1976).

[b18] Jiang X. *et al.* Highly efficient nanoarchitectured Ni_5_TiO_7_ catalyst for biomass gasification. ACS Appl Mater & Inter 4, 4062–4066 (2012). 10.1021/am3008449 22780222

[b19] Tong W. M. *et al.* Kinetic Control of MnWO_4_ Nanoparticles for Tailored Structural Properties. J. Phys. Chem. C 114, 15298–15305 (2010).

[b20] Bayati M. R., Moshfegh A. Z. & Golestani-Fard F. Synthesis of narrow band gap (V_2_O_5_)_x_–(TiO_2_)_1−x_ nano-structured layers via micro arc oxidation. Appl Surf Sci 256, 2903–2909 (2010).

[b21] Yerokhin A. L., Nie. X., Leyland A., Matthews A. & Dowey S. J. Plasam electrolysis for surface engineering. Surf Coat Technol 122, 73–93 (1999).

[b22] Sarbishei S., Sani M. A. F. & Mohammadi M. R. Study plasma electrolytic oxidation process and characterization of coatings formed in an alumina nanoparticle suspension. Vacuum 108, 12–19 (2014).

[b23] Bayati M. R., Golestani-Fard F. & Moshfegh A. Z. Photo-Degradation of Methelyne Blue over V_2_O_5_–TiO_2_ Nano-Porous Layers Synthesized by Micro Arc Oxidation. Catal Lett 134, 162–168 (2009).

[b24] Yerokhin A. L., Nie X., Leyland A. & Matthews A. Characterisation of oxide films produced by plasma electrolytic oxidation of a Ti-6Al-4V alloy. Surf Coat Techn 130, 195–206 (2000).

[b25] Durdu S., Deniz Ö. F., Kutbay I. & Usta M. Characterization and formation of hydroxyapatite on Ti6Al4V coated by plasma electrolytic oxidation. J Alloy Compd. 551, 422–429 (2013).

[b26] Albella J. M., Montero I. & Martinez-Duant J. M. Electron injection and avalanche during the anodic oxidation of tantalum. Solid-State Sci and Techn. 131, 1101–1104 (1984).

[b27] Xu X. J., Fu Q., Guo X. G. & Bao X. H. A highly active "NiO-on-Au" Surface Architecture for CO Oxidation. ACS Catal. 3, 1810–1818 (2013).

[b28] Bamwenda G. R., Tsubota S., Nakamura T. & Haruta M. The influence of the preparation methods on the catalytic activity of platinum and gold supported TiO_2_ for CO oxidation. Catal Lett 44, 83–87 (1997).

[b29] Mahammadunnisa S., Manoj Kumar Reddy P., Lingaiah N. & Subrahmanyam C. NiO/Ce_1−x_Ni_x_O_2−δ_ as an alternative to noble metal catalysts for CO oxidation. Catal. Sci. Technol 3, 730–736 (2013).

[b30] Grillo F., Natile M. M. & Glisenti A. Low temperature oxidation of carbon monoxide: the influence of water and oxygen on the reactivity of a Co_3_O_4_ powder surface. Appl Catal B: Environ 48, 267–274 (2004).

[b31] Ren Z. *et al.* Monolithically Integrated Spinel M_x_Co_3-x_O_4_ (M=Co, Ni, Zn) Nanoarray Catalysts: Scalable Synthesis and Cation Manipulation for Tunable Low-Temperature CH_4_ and CO Oxidation. Angew Chem Int Edit 53, 7223–7227 (2014). 10.1002/anie.201403461 24890371

[b32] Liang J., Srinivasan P. B., Blawert C., Störmer M. & Dietzel W. Electrochemical corrosion behaviour of plasma electrolytic oxidation coatings on AM50 magnesium alloy formed in silicate and phosphate based electrolytes. Electrochim Acta 54, 3842–3850 (2009).

[b33] Zheng H. Y., Wang Y. K., Li B. S. & Han G. R. The effects of Na_2_WO_4_ concentration on the properties of microarc oxidation coatings on aluminum alloy. Mater Lett 59, 139–142 (2005).

