# Supplementary material for: Size-controllable Ni5TiO7 nanowires as promising catalysts for CO oxidation
Source: Sci Rep. 2015 Sep 23;5:14330. doi: 10.1038/srep14330 (PMC4585801; doi:10.1038/srep14330)
Supplement: Supplementary Information [file srep14330-s1.pdf]

Supporting information

Size-controllable Ni_5TiO_7 nanowires as promising catalysts for CO oxidation

Yanan Jiang,[1] Baodan Liu,[1,*] LiniYang,[2] Bing Yang,[1]Xiaoyuan Liu,[1] Lusheng Liu,[1] Christian Weimer,[3] Xin jiang[1,*]

[1] Shenyang National Laboratory for Materials Science, Institute of Metal Research (IMR), Chinese Academy of Sciences (CAS), No. 72 Wenhua Road, Shenyang 110016 China

[2]College of Chemistry, Liaoning University, Shenyang, Liaoning, 110036, China

[3]Institute of Materials Engineering, University of Siegen, Paul-Bonatz-Straße 9-11, Siegen, 57076 Germany

Keywords: Ni_5TiO_7; nanowires; synthesis; size controlling; CO oxidation

*To whom correspondence should be addressed: baodanliu@hotmail.com

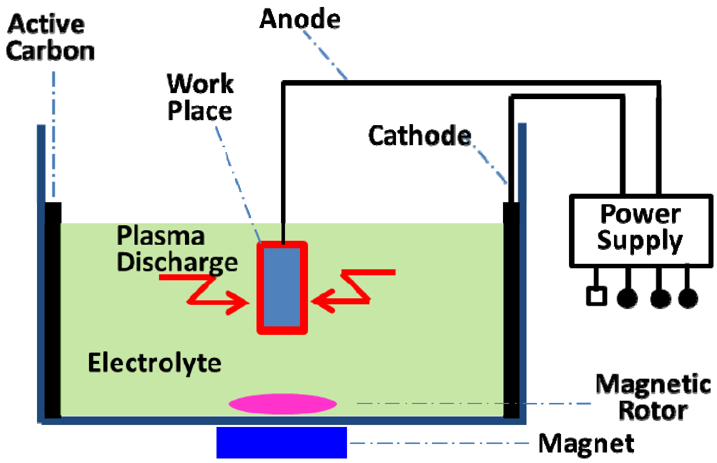

Figure S1 Schematic diagram of the experimental setup for PEO coating

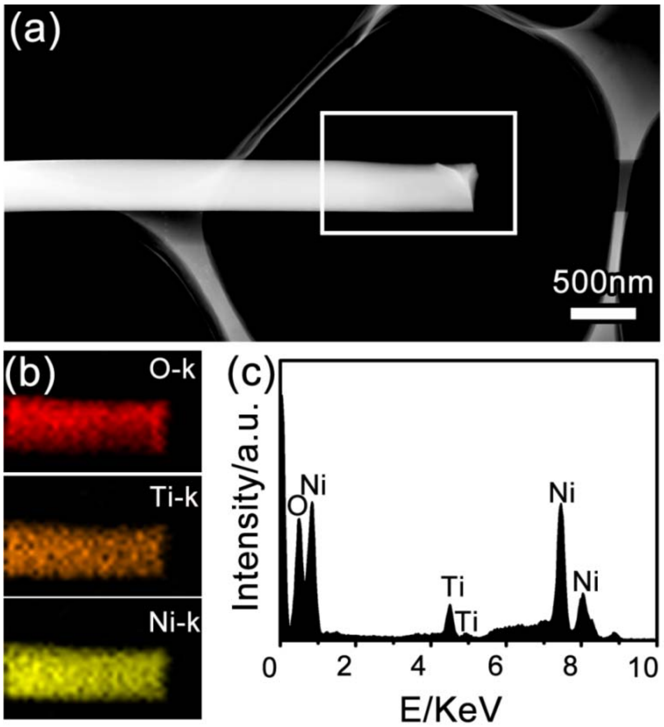

Figure S2 (a) typical TEM image of Ni_5TiO_7 nanowire; (b) corresponding TEM-EDS element mappings of O, Ti and Ni; (c) corresponding EDS spectrum of Ni_5TiO_7 nanowire.

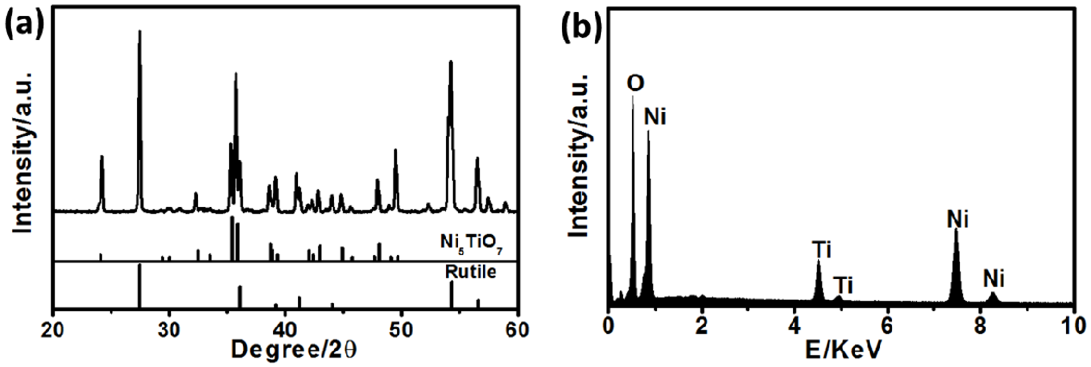

Figure S3 (a) XRD pattern of the PEO coating annealed at 1050°C without dipping in $Ni(NO_3)_2$ solution and (b) its corresponding EDS spectrum, which shows the ingredient elements of Ni, Ti and O..
